# Route of Infection in Melioidosis

**DOI:** 10.3201/eid1104.041051

**Published:** 2005-04

**Authors:** Jodie L. Barnes, Natkunam Ketheesan

**Affiliations:** * James Cook University, Townsville, Queensland, Australia

**Keywords:** Burkholderia pseudomallei, melioidosis, virulence

**To the Editor:** Melioidosis is an emerging tropical infectious disease, the incidence of which is unknown in many developing countries because of the lack of diagnostic tests and medical practitioners' lack of awareness of the disease. It is a potentially fatal disease caused by the soil bacterium *Burkholderia pseudomallei*. Clinical manifestations, severity, and duration of *B. pseudomallei* infection vary greatly (*1*).

Melioidosis develops after subcutaneous infection, inhalation, or ingestion of contaminated particles or aerosols. Infection has occurred after near-drowning accidents (*1**–**3*) and transmission of *B. pseudomallei* in drinking water (*4*). The route of *B. pseudomallei* infection is at least 1 of the factors that influences disease outcome, thus contributing to the broad spectrum of clinical signs associated with melioidosis. Researchers use different routes of delivery of *B. pseudomallei* in experimental models to study the pathogenesis of the disease and the induction of host protection. Infection by different routes exposes a pathogen to different components of the host immune system and may subsequently influence disease outcome. Despite this difference, no comprehensive investigation has compared the pathogenesis of melioidosis established by different routes of infection.

Following intravenous (IV) injection, BALB/c mice are highly susceptible, and C57BL/6 mice are relatively resistant to *B. pseudomallei* infection (*5*). Using this murine model, we compared the pathogenesis of *B. pseudomallei* infection after introducing the bacterium by IV, intraperitoneal (IP), intranasal, oral, and subcutaneous (SC) routes of infection. The virulence of 2 *B. pseudomallei* strains (NCTC 13178 and NCTC 13179) was compared in BALB/c and C57BL/6 mice by using a modified version of the Reed & Meunch (1938) method. Compared to BALB/c mice, C57BL/6 mice are less susceptible to *B. pseudomallei* infection, regardless of the portal of entry, thus validating the model of differential susceptibility for various routes of infection (Table). However, as demonstrated by others (*5**–**7*), C57BL/6 mice are not completely resistant to infection by *B. pseudomallei*. Systemic melioidosis can be generated in C57BL/6 mice by using different routes of infection, if a high dose is used. When injected IV into BALB/c mice, NCTC 13178 is highly virulent since the 50% lethal dose (LD_50_) is <10 CFU. However, if BALB/c mice are injected SC with NCTC 13178, the LD_50_ value increases 100-fold to 1 x 10^3^ CFU. This value is equivalent to the LD_50_ of the less virulent NCTC 13179 delivered SC The results emphasize that virulence depends on the route of infection.

**Table:** Ten-day LD_50_* values (given in CFU) after intravenous, intraperitoneal, subcutaneous, intranasal, or oral introduction of NCTC 13178 or NCTC 13179 strains of *Burholderia pseudomallei* into BALB/c or C57BL/6 mice

Route of infection	NCTC 13178		NCTC 13179	
	BALB/c	C57BL/6	BALB/c	C57BL/6
Intravenous	<10	5 x 10^3^	9 x 10^3^	6 x 10^6^
Intraperitoneal	1.2 x 10^1^	9.7 x 10^3^	4.7 x 10^5^	2.1 x 10^7^
Subcutaneous	1 x 10^3^	9 x 10^5^	9 x 10^2^	>10^8^
Intranasal†	1.4 x 10^2^	1.8 x 10^3^	1.9 x 10^6^	>10^8^
Oral‡	7.2 x 10^3^	1.8 10^6^	4.8 x 10^6^	>10^8^

*50% lethal dose. †20 µL of challenge dose was introduced intranasally onto the nostrils of the mice by using a pipette tip. ‡20 µL of challenge dose was introduced orally to the back of the throat of the mice by using a pipette tip.

The pathogenesis of *B. pseudomallei* NCTC 13178 infection was compared after infection by the IV, IP, SC, intranasal, and oral routes. BALB/c and C57BL/6 mice were administered 570 CFU (equivalent to 60 x LD_50_ delivered IV) or 3 x 10^5^ CFU (equivalent to 60 x LD_50_ delivered IV), respectively. At 1, 2, and 3 days postinfection, bacterial loads were measured in blood, spleen, liver, lungs, lymph nodes (right and left axillary and inguinal), and brain by using methods described previously (*5*).

A tropism for spleen and liver was demonstrated following infection by each of the 5 routes. *B. pseudomallei* could be detected in the tissues of IV- and IP-infected mice earlier and in higher numbers than in those of intranasally and orally-infected mice, despite the fact that all mice received equal numbers of bacteria. This finding reflects differences in the innate immune response, depending on the route of infection. Bacterial numbers in mice infected by the IV or IP route reached >10^6^ CFU by day 2 postinfection, which indicates a failure of the innate immune response to control infection, leading to overwhelming sepsis and death.

Bacterial loads in tissues after challenge with a lethal dose of highly virulent NCTC 13178 did not indicate any tropism for the lung after intranasal infection. As early as day 1, bacterial loads were greatest in the liver and spleen, not lungs, of C57BL/6 and BALB/c mice following intranasal challenge. This finding suggests a very early systemic spread of *B. pseudomallei* from the lungs to other organs.

Bacteria were detected in the brains of all mice after infection by either the IV, IP, intranasal, or oral route. Colonies recovered from the brains of C57BL/6 mice infected by the intranasal or oral routes were mucoid in appearance. In comparison, bacteria recovered from brains of C57BL/6 mice that were challenged by the IV or IP route demonstrated the characteristic wrinkled shape on Ashdown agar and may have been a consequence of the overwhelming septicemia that spilled over to all organs. Variation in colonial morphology of *B. pseudomallei* has been documented previously (*8*), and biofilm formation may be an adaptation of *B. pseudomallei* that enables it to evade host immune responses or to survive within unfavorable environments (*9**,**10*). The variation in colonial morphology on Ashdown agar observed in bacteria isolated from brains of C57BL/6 mice infected by the intranasal or oral route may reflect a change to biofilm formation of *B. pseudomallei* in this tissue.

In summary, the results of this study reiterate the validity of the mouse model for differential susceptibility to *B. pseudomallei*, regardless of the route of infection. The data also emphasize that virulence depends on the portal of entry of *B. pseudomallei*. Researchers should, therefore, be particularly cautious when comparing and extrapolating data from studies that use different methods of infection.
